# Improved accelerated breath-hold radial cine image reconstruction by acquiring additional free-breathing data between breath-holds

**DOI:** 10.1186/1532-429X-14-S1-P269

**Published:** 2012-02-01

**Authors:** Seunghoon Nam, Mehmet Akcakaya, Yongjun Kwak, Beth Goddu, Kraig V Kissinger, Warren J Manning, Vahid Tarokh, Reza Nezafat

**Affiliations:** 1 Medicine, Beth Israel Deaconess Medical Center, Boston, MA, USA; 2 Radiology, Beth Idrael Deaconess Medical Center, Boston, MA, USA; 3 School of Engineering and Applied Sciences, Harvard University, Cambridge, MA, USA

## Background

Evaluation of cardiac function is clinically performed using multi-slice breath-hold (BH) acquisition, in which patient breathes for a period of 40-60 seconds between a BH of 10-12 seconds. This results in suboptimal data acquisition efficiency. In this study, we propose to take advantage of the time between multiple BHs to acquire additional free-breathing (FB) data that can be used in the reconstruction of undersampled BH acquisition without increasing the total scan time.

## Methods

**Figure 1:**
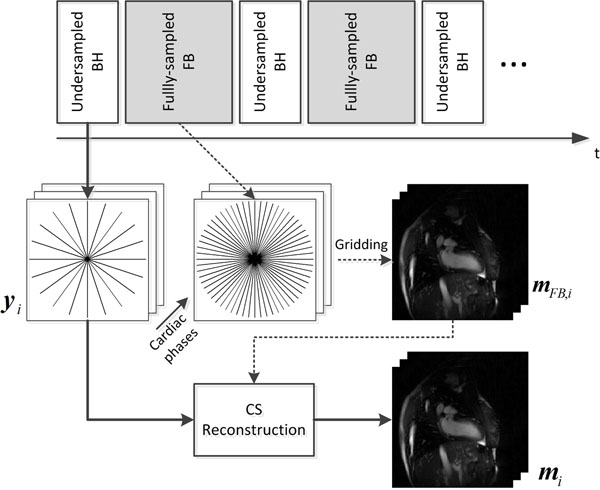
Acquisition of BH and FB radial cine data and the CS reconstruction.

Figure 1 shows the proposed data acquisition strategy. An undersampled BH acquisition is followed by a fully-sampled FB acquisition during the resting period between BH acquisitions when the patients were instructed to breathe normally. The undersampled radial cine data were reconstructed using compressed sensing (CS) [1]. Each cardiac phase image was sparsified by subtracting the FB image with the same cardiac phase for successful CS reconstruction. Each cardiac phase image is reconstructed by an iterative CS algorithm which minimizes the objective function ∥***Am****_i_*-***y_i_***∥_2_^2^+*λ*_1_∥**Ψ*m****_i_*∥_1_+*λ*_2_∥***m****_i_*-***m***_FB,_*_i_*∥_1_, where ***m****_i_* denotes the *i*-th cardiac phase image, ***y****_i_* denotes the undersampled radial k-space measurement for *i*-th cardiac phase, ***A*** is the radial acquisition matrix, **Ψ** is a sparsifying transform matrix, and ***m***_FB,_*_i_* is the *i*-th cardiac phase image from the fully-sampled FB acquisitions. The fully-sampled FB images are reconstructed by gridding algorithm [2]. The data were acquired on 1.5T Philips scanner with ECG-gated SSFP sequence using 5-channel cardiac-coil array. The following parameters were used: FOV=(320mm)^2^, spatial resolution=(1.7mm)^2^, TR/TE/α=3.1/1.5/55°, temporal resolution=34ms. All BH and FB acquisitions were acquired fully-sampled, and the BH acquisition was retrospectively undersampled. Four FB data set were acquired for each BH dataset and averaged to get ***m***_FB,_*_i_*.

## Results

**Figure 2:**
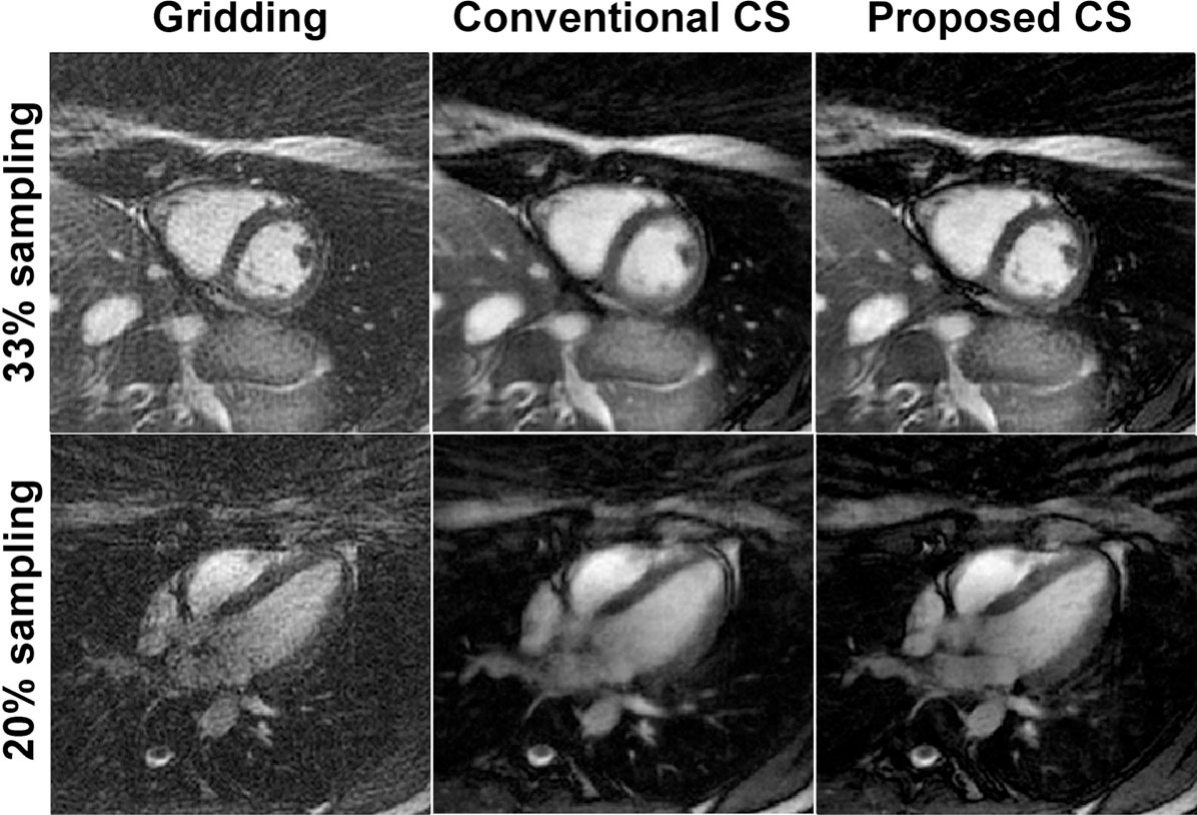
Example cardiac phase images of 33% and 20% sampling. The proposed CS reconstructions exhibit less streaking artifacts, improved sharpness and improved image qualities.

Figure 2 shows example cardiac phase images reconstructed by the proposed CS algorithm, conventional CS algorithm which utilizes *x*-*f* space sparsity [3], and the gridding algorithm. The BH data was undersampled to have 33% and 20% of projections from fully-sampled data. The proposed method has less streaking artifacts, sharper blood-myocardium borders and improved image qualities.

## Conclusions

We demonstrated the feasibility of CS reconstruction for undersampled radial cine imaging utilizing the FB data acquired during the resting period between BH acquisitions. The results show that the CS reconstruction improves the image quality and can be used either to reduce the BH duration or increase the spatio-temporal resolution of cine imaging. Further studies to evaluate global cardiac function in patients are needed to validate the clinical accuracy of the proposed technique.

## Funding

NIH R01EB008743-01A2.
